# The utilization of small non‐mammals in traumatic brain injury research: A systematic review

**DOI:** 10.1111/cns.13590

**Published:** 2021-02-04

**Authors:** Nurul Atiqah Zulazmi, Alina Arulsamy, Idrish Ali, Syafiq Asnawi Zainal Abidin, Iekhsan Othman, Mohd. Farooq Shaikh

**Affiliations:** ^1^ Neuropharmacology Research Laboratory Jeffrey Cheah School of Medicine and Health Sciences Monash University Malaysia Selangor Darul Ehsan Malaysia; ^2^ Department of Neuroscience Central Clinical School The Alfred Hospital Monash University Melbourne Vic. Australia; ^3^ Liquid Chromatography Mass Spectrometry (LCMS) Platform Jeffrey Cheah School of Medicine and Health Sciences Monash University Malaysia Selangor Darul Ehsan Malaysia

**Keywords:** animal model, differential method, non‐mammals, traumatic brain injury

## Abstract

Traumatic brain injury (TBI) is the leading cause of death and disability worldwide and has complicated underlying pathophysiology. Numerous TBI animal models have been developed over the past decade to effectively mimic the human TBI pathophysiology. These models are of mostly mammalian origin including rodents and non‐human primates. However, the mammalian models demanded higher costs and have lower throughput often limiting the progress in TBI research. Thus, this systematic review aims to discuss the potential benefits of non‐mammalian TBI models in terms of their face validity in resembling human TBI. Three databases were searched as follows: PubMed, Scopus, and Embase, for original articles relating to non‐mammalian TBI models, published between January 2010 and December 2019. A total of 29 articles were selected based on PRISMA model for critical appraisal. Zebrafish, both larvae and adult, was found to be the most utilized non‐mammalian TBI model in the current literature, followed by the fruit fly and roundworm. In conclusion, non‐mammalian TBI models have advantages over mammalian models especially for rapid, cost‐effective, and reproducible screening of effective treatment strategies and provide an opportunity to expedite the advancement of TBI research.

## Graphical abstract

Traumatic brain injury (TBI) perpetuates lifelong and dynamic effects on health and well‐being. Most of the animal models previously developed to study the TBI pathophysiology were focused mainly on mammalian animal model. Despite of all the contributions that they offered, these mammalian animal models have some drawback such as huge sample size needed and very costly which resulted in lengthy process of preclinical development phase with hindered reproducibility option. Hence, this systematic review gathered all the previous non‐mammalian animal model used in TBI research from January 2010 to December 2019 via 3 search engines which were Embase, Scopus, and PubMed. PRISMA method was chosen to select the research article for the discussion. The result shown on 3 popular species comprised of *Danio rerio* (zebrafish), *Drosophila melanogaster* (fruit fly), and *Caenorhabditis elegans* (roundworm). Criteria that were discussed were mainly on type of animal model, the functional findings, pathophysiological outcomes, and the limitation of the respective models. In conclusion, this non‐mammalian animal model provides more simplistic approach to bridge the knowledge gaps within TBI research and may shorten the preclinical process for the final findings on the outcome prevention and cure.

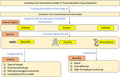

## 1 INTRODUCTION

Traumatic brain injury (TBI) is one of the leading causes of death and morbidity worldwide especially in industrialized countries.^1^ TBI has become a major public health concern with a global prevalence that has escalated to almost 27.08 million people in 2016 as reported by the Global Burden of Diseases, Injuries, and Risk Factors (GBD) study.^2^ The study also stated that about 8.1 million people were living with long‐term disability caused by TBI, mainly due to falls and motor vehicle accidents.

Traumatic brain injury has also been alarmingly related to a number of adverse long‐term effects, including elevated risk toward long‐term complications such as Parkinson's disease, Alzheimer's disease, Dementia Pugilistica, and posttraumatic epilepsy.^3^ TBI is comprised of two phases which are the primary and secondary injury phase. The primary injury phase is the initial impact encountered from the external mechanical force that results in blood vessel damage, axonal tearing,^4^ cell death at the injury site, blood‐brain barrier disruption, presence of edema, and generation of damage‐associated molecular patterns (DAMPs).^5^ Consecutively, these immediate primary injury events lead to the later secondary phase of injury comprised of glutamate excitotoxicity, mitochondrial dysfunction, and neuroinflammation. Hence, understanding this multifactorial disease by employing these animal models as well as determining its therapeutic timeframe has been a major goal of TBI research, which could be best achieved through preclinical animal studies.

Preclinical animal models have been used for decades to answer questions relating to the human condition. Preclinical TBI studies to date have heavily depended on the usage of mammalian models due to its close anatomical and physiological resemblance to humans. Rodent models, large mammalian models (pig and sheep) and non‐human primate models have successfully elucidated some of the cellular and molecular aspects of human TBI,^6^, ^7^, ^8^ the disadvantage on using these mammalian models include time^9^ and cost,^10^ resulting in the lengthy and expensive preclinical development phase for new therapeutic options. Therefore, with the growing demand for TBI preclinical research and treatment screening, a more effective animal model should be utilized instead.

Emerging TBI preclinical research reported on non‐mammalian animal models which includes zebrafish (*Danio rerio*), fruit fly (*Drosophila melanogaster*) and roundworm (*Caenorhabditis elegans*).^11^, ^12^, ^13^ This is in light of recent research that suggests nearly 70% of disease‐related genes in humans can be found in zebrafish and fruit flies, while roundworms possess about 40% of these genes,^14^, ^15^, ^16^ thus allowing for a significant range of TBI‐related long‐term disease outcomes to be studied. The growing interest in non‐mammalian preclinical models is highly desirable as these animal models are much simpler in physiology for target investigations as well as allow for rapid, cost‐effective, and highly reproducible research. Although these animal models are of different species, they still closely resemble human TBI pathophysiology and thus provide a great tool for preliminary investigations and high‐throughput screening before launching in to more detailed and comprehensive evaluations in mammalian models.

Therefore, this systematic review aims to summarize, elucidate, and critically analyze the utilization of common non‐mammalian preclinical TBI models in traumatic brain injury research, which may provide some interesting but crucial insight into the TBI pathology that resembles human TBI, and thus improve future research on therapeutic intervention for TBI patients.

## 2 METHODOLOGY AND SEARCH STRATEGY

### 2.1 Search strategy

The literature search was focused on studies published between January 2010 and December 2019. This time frame was specified to ensure that we retrieve only the most relevant recent literature.^17^ The initial search was conducted using three databases which were PubMed, SCOPUS, and EMBASE. Search terms such as “brain injury,” “traumatic brain injury” and “TBI” were first performed on the databases to create an initial list of all the relevant TBI articles. This list of articles was then sieved through and the non‐mammalian animal models were categorized according to species. An initial search resulted in the identification of the common non‐mammalian models used in the majority of the TBI studies which were only these 3 species: zebrafish, roundworm, and fruit fly. Hence, the main keywords such as “zebrafish,” “fruit fly,” and “roundworm” with their respective scientific names such as “*Danio rerio*,” “*Drosophila melanogaster*” and “*Caenorhabditis elegans*” was performed and all the relevant articles were downloaded. Next, all the titles and abstracts were screened according to the inclusion and exclusion criteria before the full article evaluation.

### 2.2 Selection criteria

Studies were selected based on the following inclusion criteria; (1) original research articles within the specified publication date range, and (2) articles which provide sufficient information on the non‐mammalian animal TBI model utilized, enabling effective evaluation and comparison for this review. The following exclusion criteria were also applied; (1) non research publications or publication with incomplete information such as symposiums, conferences, editorials, book chapters, reviews, and systematic reviews, (2) duplicated articles, (3) non‐English articles, and (4) articles that were not related to non‐mammalian animal models used in traumatic brain injury research where they did not specifically mention the usage of non‐mammalian animal model in traumatic brain injury research. The study selection for this review was performed based on the Preferred Reporting Items for Systematic reviews and Meta‐Analyses guidelines (PRISMA).^18^

The quality of the articles included in this review was assessed using the SYstematic Review Centre for Laboratory animal Experimentation Risk of Bias (SYRCLE RoB tool)^19^ (Table S1).

## 3 RESULTS AND DISCUSSION

**FIGURE 1:**
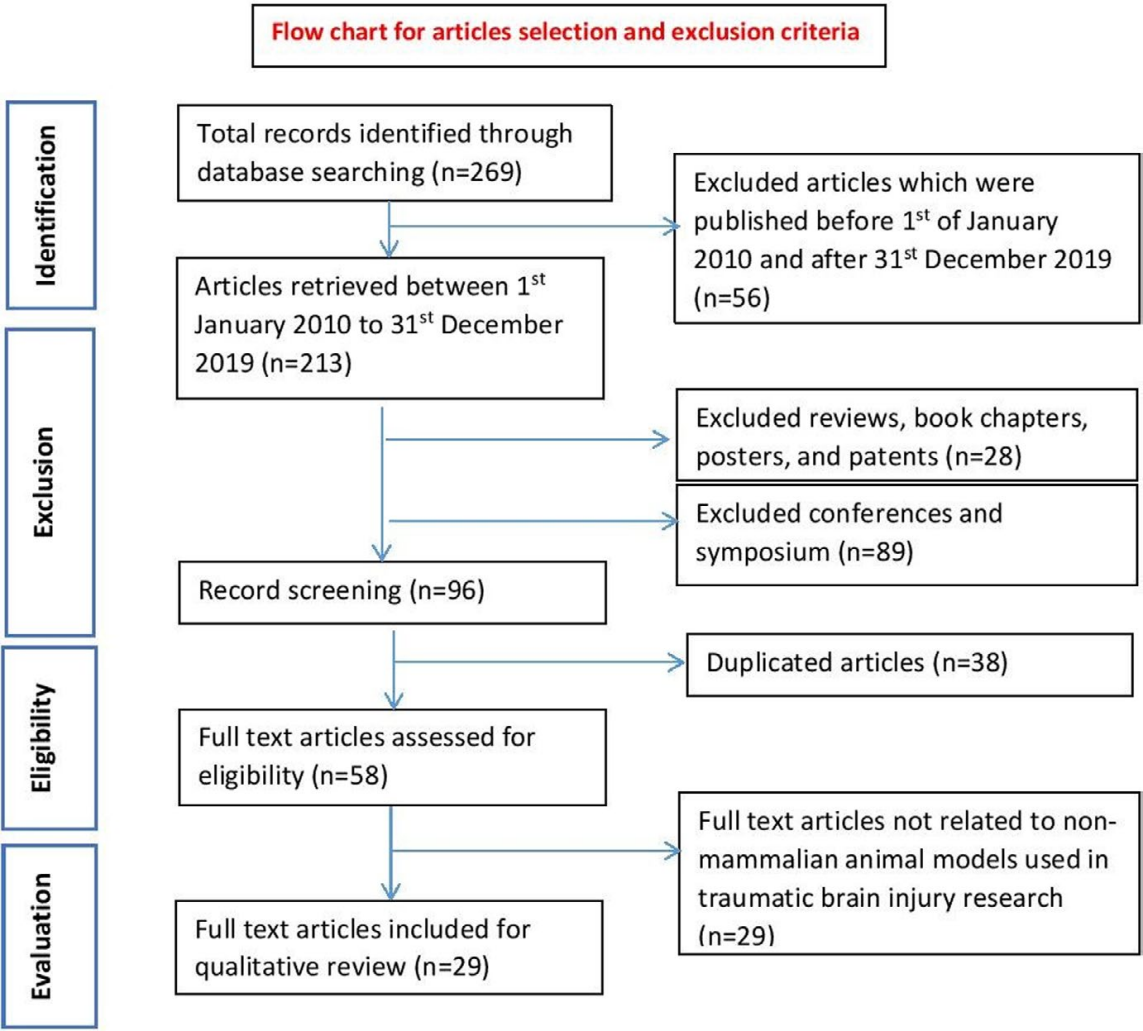
Flow chart of study selection based on the PRISMA guidelines

The literature search yielded a final total of 269 articles. After applying the inclusion and exclusion criteria, a total of 240 articles were excluded which included; (a) 56 articles not published within the specified date range (1^st^ January 2010 – 31^st^ December 2019), (b) 117 non‐original research articles, (c) 38 duplicates, and (d) 29 articles that were not related to non‐mammalian animal models of traumatic brain injury (Figure 1). Hence, the final total of full‐text articles included for critical appraisal in this review was 29 studies, which can be found compiled in Table 1. Among these articles, three articles examined TBI in roundworm, eight articles in the fruit fly, and the other 18 in the zebrafish.

**TABLE 1:** Summary of the selected articles on non‐mammalian TBI models

Animal model	Scientific name (strain) (age)	Injury model	Functional findings	Pathophysiological outcomes	Limitations of the model described	References
Roundworm	*Caenorhabditis elegans* (Wild type N2)	Therapeutic shock wave device	Dose‐dependent reduction in mean speed of worm movement Dose‐dependent increase in percentage of worms rendered paralyzed	Not applicable	Strains raised in different agar plates exhibited different speed of movement and the heterogeneity of the worms at baseline	Angstman et al.^20^
	*Caenorhabditis elegans* (Wild type N2) (Mutant type DR26 daf‐16(m26) (Mutant type CB1370 daf‐2(e1370)	Therapeutic shock wave device	Reduction of lifespan after the shockwave seen with all the strains NGM agar plates worms exhibited shorter lifespan with 500 shock wave compared to 100 shock wave Cavitation effect attenuated by PVA resulted in significant longer lifespan	Not applicable	Not applicable	Angstman et al.^21^
	*Caenorhabditis elegans* (Wild type N2)	High‐frequency acoustic wave (SAW)	Learning delays observed at lower SAW intensity and paralysis observed at higher SAW intensity	Not applicable	Not applicable	Miansari et al.^22^
Fruit fly	*Drosophila melanogaster* (white (w^1118^) flies) (0–3 day old)	High‐Impact trauma (HIT)	Temporary disability and impaired movement observed Mortality increased after injury 42 fly lines showed variation in mortality (MI_24_) after primary injury threshold	Innate immune response activation resembling the secondary injury mechanism Neurodegeneration seen	Not applicable	Katzenberger et al.^23^
	Drosophila melanogaster (w^1118^ flies) (0–7 or 20–27‐day old)		The mortality index at 24 h (MI_24_) after injury showed higher in older groups and genotype dependent	Not applicable	Tendency of inconsistency of unskilled person in charge in the preparation of the device will lead to inconsistent outcome	Katzenberger, Loewen, et al.^24^
	*Drosophila melanogaster* (Genotype w1118 (BL 5905) and y1w1 (BL 1495))		Injury severity is proportional to the deflection angle and higher severity resulted in higher in mortality Locomotor abilities reduced after the injury but recovered to normal state at 24 h post‐TBI	Not applicable	Not applicable	Putnam et al.^25^
	*Drosophila* Genetic Reference Panel (DGRP)		Not applicable	Age and diet influenced the mortality through different secondary injury pathways	Not applicable	Katzenberger et al.^26^
	Drosophila melanogaster (male flies that are cultured as previously described in ref. 27)	Blast simulator	Disruption of motor function 24 h in 5 days post‐blast period and full recovery at 8 days post‐injury The lifespan and mortality of flies decreased despite the recovery of motor function	Not applicable	Not applicable	Hockey et al.^28^
	Drosophila melanogaster (Canton‐S WT female flies) (2‐day‐old)	Blast strike	Walking ability and distance travel after injury reduced Mobility recovered within 4 min and walking activity recovered after 2 days after 1 strike at 5.0 L/min Lifespan reduced with increase in flow rate of the strike	Not applicable	Not applicable	Sun and Chen^29^
	*Drosophila melanogaster* (w^1118^ males) (NF‐κB Relish null mutants) (RepoGal4) (UAS GFP:RpL10A) (3–7 days old)	Drosophila Closed Head Injury (dCHI) unanesthetized	Mortality increased dose‐dependent in 24 h after injury Negative geotaxis impaired after 5–10 consecutive hits in dose‐dependent manner Sleep pattern decreased after injury	Innate immune response activation after injury Apoptosis seen within first 24 h	Not applicable	van Alphen et al.^30^
	*Drosophila melanogaster* (Canton‐S, w1118) (APPL‐Gal4) (PUAS‐Tau.wt1.13 (BL#−51362, G. Jackson))	Omni Bead Ruptor	Injury sensitivity increased	Innate immune system activation observed	Risk of intestinal barrier dysfunction Confounding effect of CO_2_ anesthesia	Barekat et al.^10^
Zebrafish	*Danio rerio* Zebrafish larvae (secA5‐YFP transgenic) (Larvae)	Larval glutamate insult	Dose‐dependent response to injury Locomotor deficits observed	Not applicable	Suitable to screen for impairment or changes to the pathophysiological processes of TBI and not represent the whole disease in its entirety	McCutcheon et al.^31^
	*Danio rerio* (Transgenic Tg(coro1a:EGFP)hkz04t and Tg(HuC:EGFP)as8) (Larvae)	Larval stab lesion	Not applicable	Microglia accumulated around the lesion after injury.	Not applicable	Gan et al.^32^
	Danio rerio (wild type: WIK strain) (Larvae)		Not applicable	Presented different phases of primary and secondary death Excitotoxicity caused secondary cell death Microglia debris clearance suggested as neuroprotective.	Not applicable	Herzog et al.^33^
	Danio rerio (macrophage‐specific lineage mpeg1:mCherry) (neutrophil‐specific mpo: GFP) (erythroid‐specific gata1:dsRed) (ubiq:secAnnexinV‐mVenus) (Larvae)	Larval intracerebral hemorrhage (ICH)	Locomotor deficits increased	Brain cell death increased Macrophage based phagocytosis observed surrounding the injury area.	Effect of edema as seen in ICH patients might not effectively presented by this type of model due to the cranium is not fully developed.	Crilly et al.^34^
	Danio rerio (wild‐type, AB strain, short fin) (adult‐ 6–12 months)	Ultrasound Injury (pHIFU)	Offered Non‐invasive method No contact model with behavioral endpoints assessed in an automated device.	Molecular changes, observed which associated with the behavioral impairments	Size ratio limitation of the pHIFU wave to only zebrafish brain Limited region‐specific analysis able for investigation limited to acute condition of the injury	McCutcheon et al.^9^
	Danio rerio (Adult)	Acoustic shock wave	50 ms shock waves at 11 MPa represented much more severe type of injury model which showed delayed recovery and displayed erratic swim patterns post‐injury.	Not applicable	Not applicable	Ferrier et al.^35^
	Danio rerio (Male wild type strain AB) (older than 90 days)	Telencephalon injury	Severe damage in tectum opticum and posterior tuberculum identified via 1325 nm 3D SD‐OCT imaging.	Not applicable	Not applicable	Zhang et al.^36^
	Danio rerio (male wild type, AB strain) (adult‐ 6‐month‐old)		Not applicable	Telencephalon exhibited strong regenerative property after injury Proliferation activity seen highest in the brain parenchyma.	Not applicable	Diotel et al.^37^
	Danio rerio (adult 5‐ to 10‐month‐old)		Not applicable	Regenerative properties seen in the injured telencephalon.	Not applicable	Kishimoto et al.^38^
	Danio rerio (Tg(kdr:EGFP)) and wildtype) (adult−6 months old)		Not applicable	Molecular restoration mechanism identified after injury.	Not applicable	Wu et al.^39^
	Danio rerio (Wild‐type AB strain) (adult‐ 5‐ to 8‐month old)		Not applicable	An important biomarker for neurogenesis identified.	Not applicable	Ayari et al.^40^
	Danio rerio (Wild‐type, gol‐b1 line in the AB strain) (adult−6–10 months old)		Not applicable	Radial glia‐type stem/progenitor cells involved in the regeneration process after a traumatic lesion Absence of permanent glial scarring seen.	Not applicable	Kroehne et al.^41^
	Danio rerio (Transgenic Tg(olig2:EGFP) Tg(GFAP:GFP), Tg(3.9nestin:GFP) Tg(Apo‐E‐GFP)) (adult−1–1.5‐year‐old)		Not applicable	Regenerative properties observed around the lesion site Oligodendrocytes increased after the lesion No permanent glial scarring observed Radial glial cells upregulated at the ventricle Moderate increase of Olig2 seen	Not applicable	März et al.^42^
	*Danio rerio* (adult)		Not applicable	Regenerative properties and gene upregulation seen around lesion	Side effect such as non‐specific cell ablation, higher cell death due to secondary degeneration, disruption and impairment of blood brain barrier, destruction of ventricular zone which allow CSF flow into brain parenchyma	Schmidt et al.^43^
	*Danio rerio* (wild‐type, AB strain) (Tg(fli1aEGFP)y1, Tg(gfap:gfp)mi2001 and Tg(olig2:gfp)) (Adult 3‐ to 4‐month old		Not applicable	Microglia seen as the earliest cell in the injury site Oligodendrocyte progenitor cells observed to be reduced after the injury The wound closed completely without any scar tissue	Not applicable	Baumgart et al.^44^
	*Danio rerio* (Tg(1016tuba1a:GFP)) (Tg(her4.1:CreERT2)) (Tg(β‐actin2:loxP‐mCherry‐loxP‐GFP)) (Tg(gfap:GFP)) (Tg(ascl1a:GFP)) (Tg(olig2:GFP) (Double transgenic Tg(her4.1:CreERT2;β‐actin2:loxP‐mCherry‐loxP‐GFP))	Quinolinic acid (QA) lesion	Not applicable	Strong neural regeneration reported and the lesion repair at the telencephalon enhanced with the QA injection	Not applicable	Skaggs et al.^45^
	*Danio rerio* (Homozygous with lofdt2, long‐fin) (Adult)	Weight drop model	Significant deficits in spatial memory test after the injury	Gene Ontology (GO) categories of peak injury pathways and neuroregeneration pathways elucidated in the zebrafish model	Not applicable	Maheras et al.^46^
	*Danio rerio* (adult)	Brain mechanical lesion	Not applicable	Characteristics of spred‐2 in the cell proliferation phase and its role in neural repair were identified after injury	Not applicable	Lim et al.^47^

Abbreviations: 3D SD‐OCT, three‐dimensional spectral‐domain optical coherence tomography; CO2, carbon dioxide; GO, gene ontology; MPa, MegaPascal; ms, millisecond; nm, nanometer; Olig2, Oligodendrocyte transcription factor; OPC, oligodendrocyte progenitors; pHIFU, Pulsed high‐intensity focused ultrasound; SAW, surface acoustic wave.

### 3.1 Roundworm (*Caenorhabditis elegans* model)

*Caenorhabditis* worm (*C*. *elegans*) is one of the least used alternate choices of animal models in TBI research (Table 1) despite the advantages it offers including the relative ease of genetic manipulation.^20^ This tiny soil nematode is known to have only 302 neurons in its entire nervous system, which is minute, compared to the 86 billion in the human brain.^21^ However, due to *C*. *elegans* possessing a very simple nervous system, high‐throughput screening or rapid trial experiments can be performed inexpensively, as compared to the mammalian model.^22^, ^23^, ^24^ Moreover, this animal model allows disease progression studies of TBI to be carried out at a quicker pace than larger animal models, as roundworm's lifespan, is anywhere between 12 to 18 days.^23^ Based on Table 1, two types of injury models have been proposed in TBI studies using the roundworm model: Therapeutic Shock Wave and High‐Frequency Acoustic Wave injury models.

#### 3.1.1 Therapeutic shock wave

This model is commonly utilized to imitate blast‐related mild traumatic brain injury (br‐mTBI) in roundworms. The shock waves generated through this injury model shared similar properties as primary blast applied in larger animal models of TBI. One of the disadvantages of using this model is the risk of developing damaging cavitation produced by the shock wave.^25^, ^26^ The modifications made by Angstman and his team have managed to reduce the cavitation by running the experiment in a low cavitation medium.^27^

**FIGURE 2:**
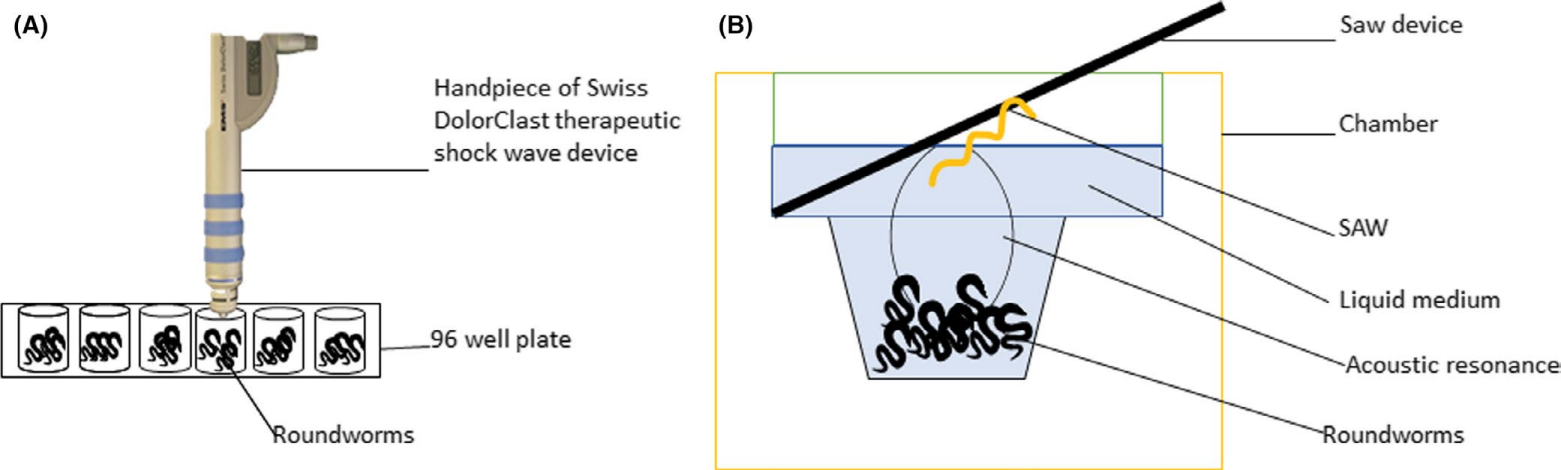
Injury models utilized by the roundworm TBI model. (A) Therapeutic shock wave applied through handpiece Swiss Dolor Clast device directly into wells containing roundworms, and (B) High‐frequency acoustic wave delivered through SAW device vertically into a liquid medium filled chamber containing roundworms

In brief, the shock wave was applied to the roundworms, contained in a medium, through the handpiece of the Swiss Dolor Clast therapeutic shock wave device (Figure 2A). This device‐generated therapeutic shock waves ballistically by speeding up a projectile that strikes an applicator and transformed the kinetic energy of the projectile into a radially expanding pressure wave. This expanding wave ensured that all the worms in the medium well were completely exposed to the wave injury.

Based on Table 1, the *C*. *elegans* exhibited severe motor deficits and recovered within 10 min following the therapeutic shock wave injury. The recovery period shown by this less complex organism is the fastest as compared to the mammalian TBI models. This injury model has fulfilled two of the criteria for blast‐related neurotrauma models,^28^ including the development of functional deficits following injury and the possibility of inducing reproducible injury in a controlled and quantifiable manner. Another significant finding observed when exerting 500 shock wave created much severe injury on NGM agar plates worms where the lifespan was much shorter. Invention was done to hinder the cavitation effect previously reported to affect nervous tissue.^25^ The blast wave's primary effect is the main damaging factor of this model and thus the molecular and cellular consequences that happened cannot be directly compared to study by^29^ on rodents model which the br‐m‐TBI caused is by blast wind as the tertiary effect. Some of the limitations of this model were observed by differences in the movement speed of the worms on different medium plates which may confound the comparison of post‐injury outcome measures across research laboratory groups.

#### 3.1.2 High‐frequency acoustic wave

This model was developed intentionally to study and imitate the blast‐related mild TBI (br‐mTBI). Previous studies showed some irregularities in the *C*. *elegans's* behavioral outcomes^27^, ^30^, ^31^ and suggested that the uncontrolled propagation, reflection, and destructive interference of the ultrasound were likely to be the root cause of the inconsistencies. Hence, modification of this injury model has adapted proper‐worm‐on‐a‐chip devices or microfluidic systems to control environmental exposure to the acoustic wave. The microfluidic system was also believed to be a great tool for studying large numbers of *C*.* elegans* at once,^32^, ^33^ thus ensuring this injury model to be highly time effective.

In brief, this injury model was induced using the surface acoustic wave (SAW) device integrated with the worm‐on‐a‐chip microfluidic system and can be performed using two techniques; by introducing the SAW‐driven acoustic waves upright into a chamber fill up with *C*. *elegans* (Figure 2B) or by preparing *C*. *elegans*, wet in sessile droplets of media and is directly placed upon the SAW device.

The exposure of the ultrasounds generated through this injury model reduces the worms mobility and causes morphological changes post‐injury.^28^ Besides that, based on Table 1, short‐term memory deficits and associative learning delays were also evident in the worms post‐injury when utilizing this TBI model.^34^ In fact, recent studies on the mechanism of *C*. *elegans* on the acoustic compressibility have been published in September 2018.^35^ In brief, this method has revealed to us that *C*. *elegans* can be used in TBI research pertaining to learning and memory study.

### 3.2 Fruit fly (*Drosophila melanogaster*)

*Drosophila melanogaster* has been utilized in numerous models of neurological disorders^36^ including models of traumatic brain injury.^37^, ^38^, ^39^, ^40^ *Drosophila* models have around 75% genetic match with humans, hence this animal model is well suited for understanding the genetic changes within the central nervous system,^38^ including as an outcome of TBI.^41^ The fruit fly consists of three regions: the protocerebrum, deutocerebrum, and tritocerebrum, which are homologous to the forebrain, midbrain, and hindbrain in humans,^42^ therefore making this non‐mammalian model a great candidate for clinically relevant, high‐throughput, fast (short lifespan), and inexpensive (massive breeding capabilities) TBI study. Four types of injury models can be performed using fruit fly: High Impact Trauma Model, Blast Model, Closed Head Injury Model, and Omni Bead Ruptor Model (Table 1).

#### 3.2.1 High‐impact trauma (HIT)

The high‐impact trauma (HIT) device was developed to study the more common closed head TBI’s underlying cellular and molecular mechanisms rather than penetrating TBI, in fruit flies.^36^ The HIT method applies mechanical force to the whole fruit fly creating widespread damage (polytrauma) including neuronal damage within the brain, which mimics the human condition, especially in motor vehicle accidents.^40^ Moreover, compared to other TBI models, the HIT model is the simplest, least costly, fastest and most amenable TBI model for fruit flies.^10^, ^39^, ^43^, ^44^, ^45^

**FIGURE 3:**
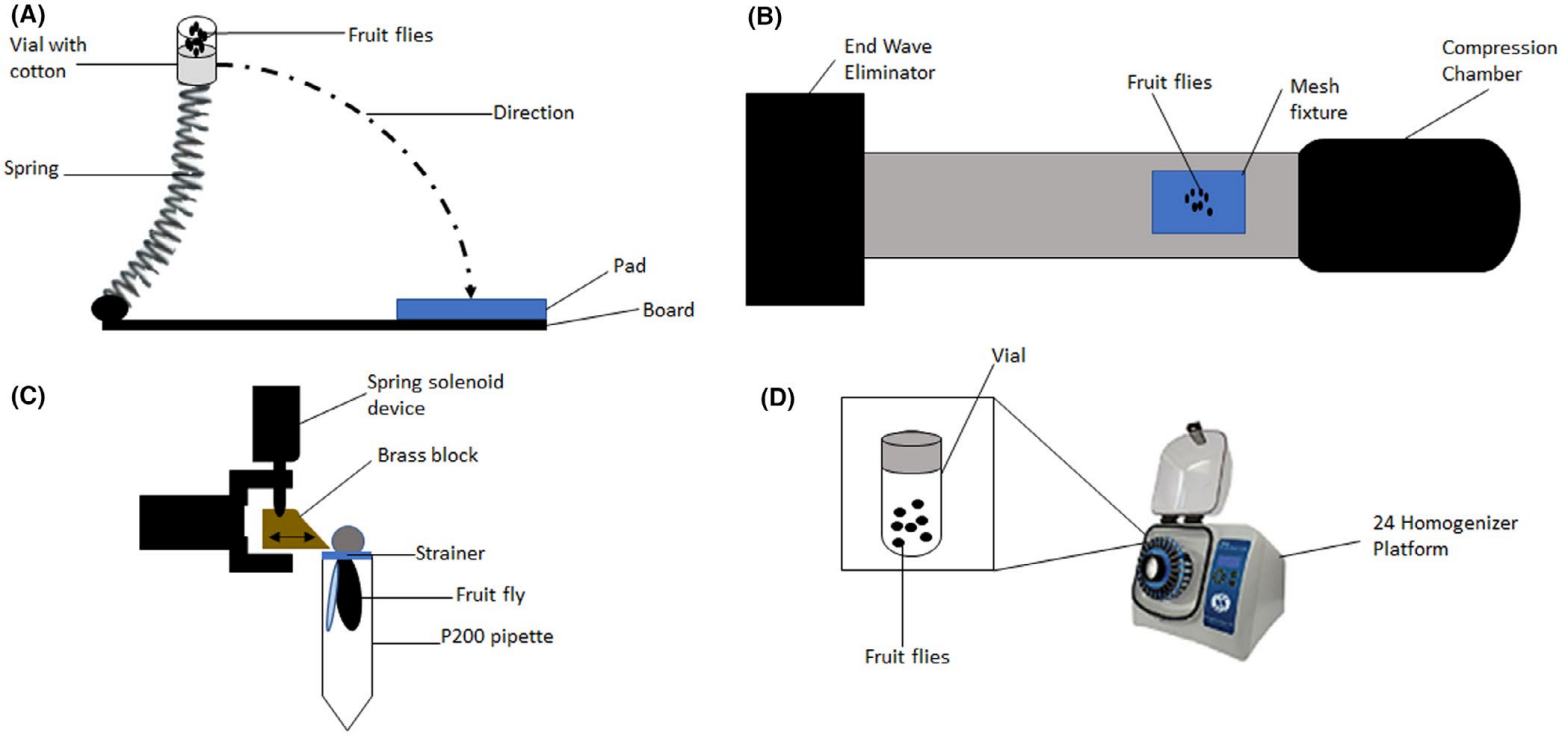
Injury models utilized by fruit fly TBI model. (A) High‐impact trauma (HIT) achieved by releasing spring attached with a vial of flies to rapidly contact the polyurethane pad, (B) Fruit flies in the mesh fixture were exposed to free‐field blast released from the compression chamber, (C) Restrained fruit flies with only the head portion exposed were impacted with solenoid recoiled brass block to achieve closed head injury, and (D) Omni Bead Ruptor‐24 Homogenizer Device with pre‐programmed shaking conditions used to deliver TBI in fruit flies contained in vials

In brief, this model involves unanesthetized or anesthetized flies confined to the bottom part of a plastic vial. The end of the vial was fitted with a stationary cotton ball. The spring with the vial attached was deflected and released thereby rapidly contacting the polyurethane pad on the bench (Figure 3A). The mechanical force was exerted on the flies as they hit the vial wall during the rebound. This injury model's advantage is that the severity of TBI can be manually adjusted either by altering the extent of spring deflection or setting the number of strikes needed for desired severity. However, this manual manipulation sometimes leads to large variability in TBI severity as each of the flies may receive different forces at once and may lead to unreliable outcomes. Thus, skilled investigators are needed to operate the HIT device so that the inconsistencies in TBI severity can be minimized. Furthermore, this model has a high mortality rate due to random injuries or polytrauma inflicted on the fruit flies,^46^ thus requires a large sample size.

Traumatic brain injury outcomes generated through this injury model were similar to closed head TBI characteristics in humans^5^, ^47^ such as temporary incapacitation ataxia, immune response activation, neurodegeneration, and death, suggesting similarities in the cellular and molecular pathological pathways between the two species. Neurodegeneration can be seen via the appearance of the vacuolar lesion in the brain neuropil, and the size is bigger in the TBI model of older age and much severe type injury model. Different strains produce a significant variation in the primary injury threshold effect which supported the previous rodent model study revealed that the TBI outcome relatively depend on genetic background.^48^, ^49^ Allen 2000 once reported in its TBI rodent model where motor deficits are less prominent in model that received severe injury after mild injury compared to the model that only received severe type of injury and support the finding that some fly lines showed low MI_24_ after exposed to 2 h inter‐injury interval compared to only 5 min interval.^50^ Interestingly, one of the studies revealed the relationship of diet that affects MI_24_ with the time interval between the first with subsequent strike onto the fly had not been studied extensively in the mammalian model. Moreover, several studies using the HIT model in fruit flies have elucidated the importance of certain factors such as age, genetic susceptibility, and the effect of multiple TBI incidents on functional outcomes and mortality after TBI, all of which have been understudied in mammalian models of TBI.

#### 3.2.2 Blast testing

Mammalian blast TBI models are often time‐consuming, expensive, and difficult to generate in large numbers.^51^, ^52^, ^53^ Thus, Drosophila's blast injury model is cost‐effective and enables high‐throughput screening may be a better alternative as a blast injury model.^38^ A blast simulator is usually a custom‐built machine consisting of a driving compression chamber, rectangular section, and end wave eliminator (Figure 3B). In experiments, fruit flies were placed in an enclosed mesh fixture at the stimulator's rectangular section. Then, free‐field blast exposure is generated at the compression chamber with an average peak overpressure of 120 kPa which the fruit flies were exposed to for a duration of 2 ms. The end wave eliminator eliminates this blast to avoid second (rebound) blast exposure in the fruit flies.^38^

This injury model displayed consistent and comparable outcomes to human mild blast TBI such as distinct motor dysfunction and high mortality rate, (Table 1). Interestingly, a modified version of this injury model enabled it to be utilized for chronic traumatic encephalopathy (CTE) studies, as it simplistically inflicted repetitive mild TBI in fruit flies.^46^ Despite showing similar injury characteristics to that of mild blast traumatic brain injury and CTE, none of the studies have attempted to reveal the underlying mechanisms related to the outcomes yet.

#### 3.2.3 Drosophila closed head injury (dCHI)

Unlike the other *Drosophila* injury models which may have shown some inconsistencies in the outcomes measured due to the whole body exertion of impact,^10^, ^54^ the closed head injury model (dCHI) minimizes the inconsistencies as the impact by focusing only toward the head of the fruit fly, thereby causing TBI without the presence of confounding peripheral injuries.^55^ Moreover, this injury model delivered precise non‐penetrating strikes to an unanesthetized fly's head, eliminating the possible confounding effects of anesthesia as implicated in mammalian TBI studies.^56^, ^57^

In brief, each fly was pushed into a P200 pipette using an aspirator and some air pressure. A specially designed strainer is then used to restrain the fly's head, thus ensuring that only the head is exposed outside the pipette. Next, using a micromanipulator, the head was positioned so that the back of the fly's head was in contact with the brass block attached to a spring‐type solenoid device (Figure 3C). The brass block then delivered force to the fly's head causing TBI. The number of hits to the fly's head can be manipulated to achieve repetitive TBI of varying severity as well.

In fact, using this injury model not only can depicted the same phenotype seen in mammalian TBI models but the usage of mutants’ species in this project showed the importance of Drosophilia genetic tools in the investigation of the novel pathway underlying TBI. Different glial genes manage to be identified in this study but most of the genes were not well understood, and it is a good opportunity for this model to serve as first‐line screening for identifying in detail the other pathways that may modulate recovery. Besides that, this model may also help to understand the genomic response during TBI and the TBI recovery pathway, at a more effective rate than mammalian TBI models.

#### 3.2.4 Omni bead ruptor

The omni bead ruptor TBI model was developed to investigate the long‐term effects of low impact or mild traumatic brain injury (mTBI).^10^ This model was designed to generate high‐throughput mTBI using the Omni Bead Ruptor‐24 Homogenizer platform in Drosophila. In brief, flies were anesthetized using CO2 exposure and were then subjected to specific pre‐programmed shaking conditions in the device (Figure 3D). In multi‐bout conditions to mimic CTE, subsequent injuries or shaking in the device were performed after the flies have recovered (usually within 30 s).

In contrast to the aforementioned HIT device,^39^ this method gave control on the impact of injury, using a programmable and automated system, therefore eliminating the potential of human error seen in the HIT device. Besides that, the mortality rate, using the Omni Bead Ruptor was relatively low compared to the HIT or blast injury model. Like the dCHI, studies utilizing this injury model in fruit flies are scarce and therefore possible limitations, besides the confounding effects of anesthesia and polytrauma, are not identified yet.

The selected study using this injury model, observed deficits in behavior and innate immune system and witnessed transformation in the profile of key autophagy markers, which were commonly described in the studies conducted in mammalian models of TBI.^58^ Hence, this Drosophila mTBI injury model will allow rapid identification and design of potential treatment options for TBI, especially targeting the molecular mechanisms revealed by the use of mammalian models, thus saving time and cost while still advancing TBI research.

### 3.3 Zebrafish (*Danio rerio*)

Zebrafish (*Danio rerio*) is one of the most common non‐mammalian animal models used in neuroscience research, even among TBI research as summarized in Table 1. Zebrafish continues to have a growing interest as an animal model due to its wide degree of genetic homology and similarities in cell signaling pathways to mammalian species^16^, ^59^ and humans.^60^ Either the adult or larvae of zebrafish can be utilized in TBI studies, therefore enabling life span/aging studies to be performed alongside TBI within a shorter timeframe, due to the shorter life span of zebrafish compared to mammalian models. Moreover, the large breeding capability from just a single adult pairing, which can yield hundreds of offspring, benefits greatly in terms of cost and time, for high‐throughput screening and preclinical research experiments.^61^ Studies using the zebrafish larvae have several advantages over the adults; these include high‐throughput screening in 96‐well plates, behavioral assessment easily performed through in‐vivo imaging, ease of application of treatments via water delivery to the wells and finally efficient absorption of compounds to the brain due to the underdeveloped blood‐brain barrier.^62^ Based on the 18 articles selected for this review (Table 1), 9 types of injury models were established for zebrafish; 3 specified for zebrafish larvae and 6 using the adult zebrafish.

#### 3.3.1 Acoustic shock wave

Similar to the shock wave principles applied in the roundworm model, this injury model inflicts mild TBI in zebrafish by mechanical stress and temporary cavitation, using fully automated acoustic shock waves, often with a 50 ms pulse length which offered much more severe injury. However, unlike the roundworms, the acoustic shock wave model in zebrafish uses confocal imaging to focus and properly align the injury toward the head of the zebrafish, thereby ensuring consistency in the inflicted TBI.

**FIGURE 4:**
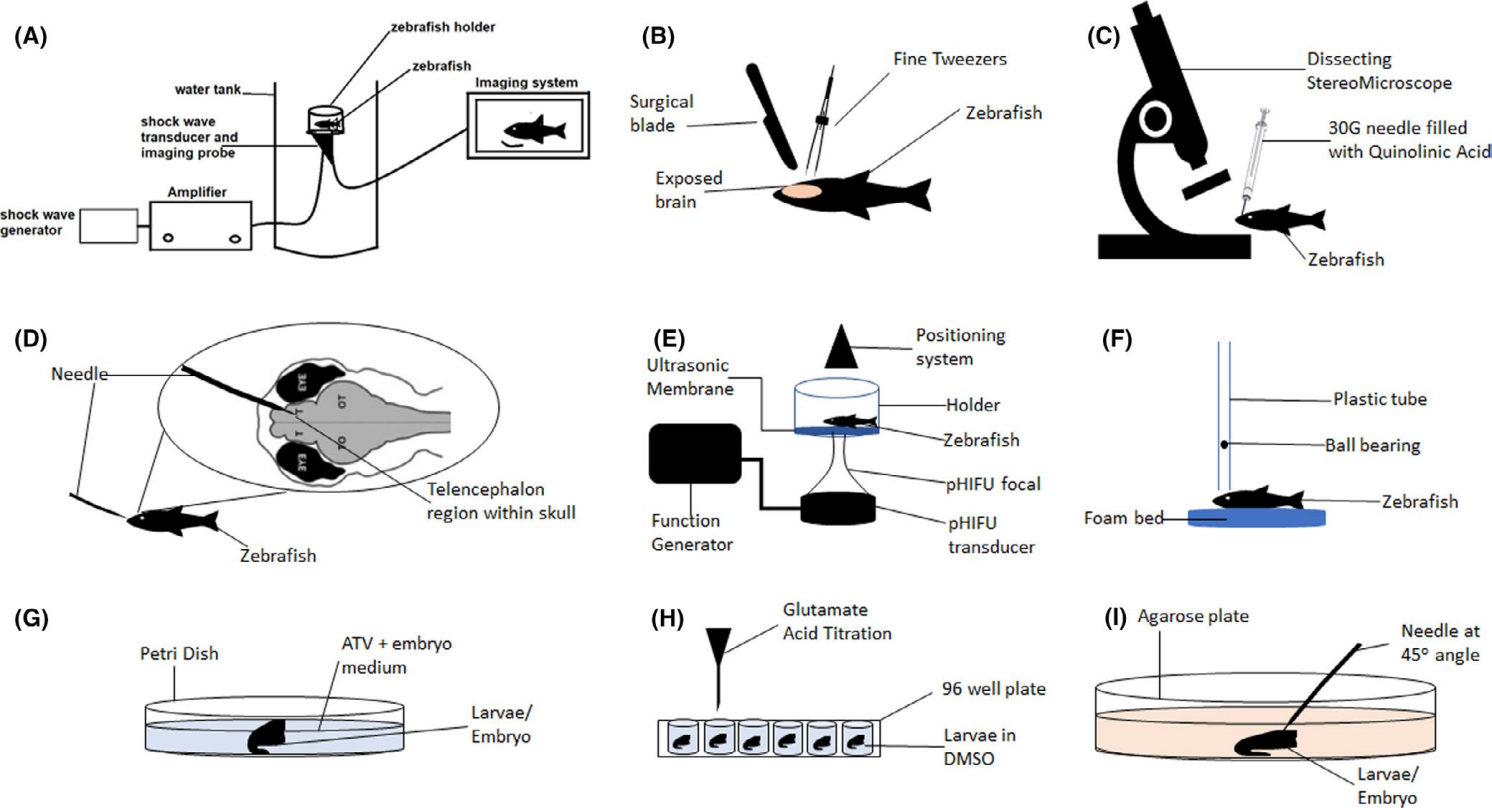
Injury models utilized by the zebrafish TBI model. (A) Acoustic shock wave generated and bombarded onto zebrafish head, directed by B‐focal imaging system, (B) Mechanical lesion applied by surgically making a hole in the skull and using the fine tweezers to cut the specified brain region, (C) Injection of quinolinic acid into desired brain region under a dissecting stereomicroscope, (D) Telencephalon injury induced by inserting needle into the telencephalon region via nose, E) Pulsed high‐intensity focused ultrasound (pHIFU) generated and focally targeted toward zebrafish head through an ultrasonic membrane, (F) A steel ball‐bearing (weight) is dropped from a specified height through a plastic tube and unto the cranium of the zebrafish, (G) Larvae was incubated in a petri dish filled with atorvastatin (ATV) and embryo medium mixture, (H) Glutamate acid was titrated into wells of a 96‐well plate containing zebrafish larvae (each in one well), and (I) Stab lesion was inflicted in zebrafish larvae placed on agarose medium, via a needle angled at 45o toward the desired brain region

In brief, zebrafish were anesthetized and placed individually in a custom‐made holder covered by a thin layer of ultrasound‐transparent mylar membrane (Figure 4A). The head of the zebrafish was tucked in properly above the membrane and the confocal B‐mode imaging was used to check the alignment. Then, the acoustic shock wave was generated and bombarded under the membrane to inflict TBI onto the zebrafish head. Besides, Linear Acoustic and Temperature simulator (LATS) program may also be used to modulate the focal acoustic intensities and shock wave pressures,^63^ giving greater control on the injury depth and severity.

Based on the selected study, zebrafish inflicted with 50 ms shock waves at 11 MPa showed a longer recovery time compared to control fish. They showed a higher anxiety effect, reduced in swimming distance and displayed irregular swimming patterns, consistent with the motor outcomes seen in mammalian models. Thus, this suggests that this injury model may be a promising tool for TBI outcome and treatment studies. Unfortunately, this injury model is still in its infancy and therefore requires further study to determine the mechanistic and pathological pathway post‐injury.

#### 3.3.2 Brain mechanical lesion

In brief, a mechanical lesion was performed by first creating a small hole on the zebrafish's head with a sterile surgical blade, and then, by using a very fine tweezer, a cut was made in the telencephalon lobe of the brain (Figure 4B). The zebrafish were left to recover after replacing the skull and closing the hole on the zebrafish head. The open head/skull or exposed brain concept as well as the targeted lesion injury principle applied through this injury model may not clinically represent human TBI, which may be a disadvantage of this injury model. Nevertheless, protein and gene expression still be accurately investigated using this model, especially when needing to investigate TBI at a specific brain region.

More studies are needed to investigate these contrasting findings between the animal models, as well as to investigate other genetic and proteomic pathways, besides spred‐2 in the neural repair process post‐injury.^64^, ^65^

#### 3.3.3 Quinolinic acid (QA) lesion

Similar to the glutamate excitotoxicity injury model, quinolinic acid (QA) is an excitotoxic metabolic agent that acts as an agonist at N‐methyl‐D‐aspartate (NMDA) receptors, therefore creating injury lesions when injected into the brain region as well as rapidly stimulates a neuroinflammatory response that promotes neuronal repair. Previously, this QA lesion injury model has commonly performed in rodent TBI models, but the extent of the neuronal repair was minimal,^66^, ^67^, ^68^, ^69^, ^70^, ^71^ unlike that observed in the adult zebrafish brain.^72^ Furthermore, the injury response with QA is more intense compared to stab injury alone, thus producing more clear and quantifiable post‐injury measures of neuronal damage and repair. Interestingly, contra‐lesion effects were also seen using this injury model in zebrafish which opens new doors toward understanding the extent of neuronal connectivity in pathological pathways post‐injury.

In brief, adult zebrafish were anesthetized and placed in a clay mold under a dissecting stereomicroscope. A 30‐gauge needle was filled up with 2.5 µl of 15 mM quinolinic acid and the needle was injected vertically through the skull into the right telencephalic hemisphere (Figure 4C) before it was placed into clean water for recovery.

The selected study in this review showed that lesion repair was strongly enhanced and more robust in the zebrafish telencephalon. Itthus proposed that QA‐induced brain lesioning model in zebrafish may provide an optimum tool to study neurodegeneration and neuronal damage replacing the less robust rodent QA lesion models. However, this model is still limited in terms of understanding the difference in molecular signals of QA lesioning compared to other excitotoxic agents, the long‐term behavioral outcomes associated with QA lesioning, and the comparison of regeneration mechanism between zebrafish and mammalian models.

#### 3.3.4 Telencephalon injury

When the stab lesion model's principles are applied to adult zebrafish with the injury site specified to the telencephalon area of zebrafish's brain, then the injury model is termed as the Telencephalon injury model. This model has been commonly used to study the neuroinflammatory response and other secondary injury pathological pathways post‐TBI. This model is unique as it is highly specific toward the telencephalon which has the capability of exhibiting strong regenerative properties after injury in zebrafish.^73^, ^74^, ^75^, ^76^, ^77^ The abundance of radial glial cells seen in the lesion area within this region was associated with the strong regenerative properties.^73^ In fact, glial restricted precursor cells, one of the glial progenitors, have been described as important candidates for repairing CNS functions such as traumatic injuries.^78^ However, the telencephalon in mammalian models lacks the radial glia cells^76^ because most of them have been converted to astrocyte in the adult brain which has a very limited capacity to regenerate depending on the severity.

Baumgart and his team reported that the injured wound was closed without any scarring and suggested that the reduction of oligodendrocyte progenitors (OPC) might be the reason underlying this regenerative phenomenon.^77^ However, more research is needed to determine the molecular mechanism governing this extraordinary feature, which may advance TBI research and other CNS disorders stemmed from neuronal cell death.

In brief, a stab lesion was performed by inserting a needle into an anesthetized zebrafish through the nostril along the rostrocaudal body axis, passing the olfactory bulb until reaching the caudal area of telencephalon (Figure 4D). The validation of this injury model was done through the histological stain of the brain with cresyl violet. The main limitation of this injury model is that it is an invasive injury model which may have certain unaccounted side effects due to the small sizes of the brain which can cause non‐specific cell ablation, increase in cell death because of secondary degeneration, the blood‐brain barrier could easily impaired and destruction of the ventricular zone. Hence, this model is perfect to represent the penetrating injury effect rather than the bump, blow or jolt type of TBI.

#### 3.3.5 Ultrasound injury (pHIFU)

High‐intensity focused ultrasound (HIFU) is the latest technology in TBI research which works by generating energy beams externally and focusing them directly on the desired injury site; the head in the case of TBI. A modified version of this model is the Pulsed HIFU (pHIFU), described as short pulses of HIFU that can induce clinically representative mechanical injury in the soft brain tissue.^79^ The HIFU transducer can generate long intensive bursts for thermal therapy or short pulses (pHIFU) for acoustic shockwave generation. This model comes with a scanner attached to capture the high‐resolution ultrasound imaging and assist in the target area's alignment. Mathematical calculation was also required by using calibrated hydrophone measurements and computer numerical simulations^80^ to estimate the acoustic focal pressure needed for the target zone.

In brief, the anesthetized fish was placed in a holder by lying on its left side and were finely secured with surgical tape. The head of the zebrafish was positioned on top of a clear ultrasonic membrane opening below the holder (Figure 4E). The pulse's amplitude and duration were set into the function generator before it was given to the zebrafish's head.

This pHIFU injury model is a non‐invasive TBI model, that focuses on a specific target region, and has an automated behavioral assessment that can be performed through video tracking software. The selected study showed that caspase 3 activity was significantly altered post pHIFU which corresponded to the deficits in behavioral outcomes,^9^ which were similar outcomes observed TBI’s mammalian models. However, the pHIFU model is limited in terms of the size ratio of the acoustic wave to the zebrafish brain, which may hinder the depth and severity of injury in TBI research.

#### 3.3.6 Weight drop model

The weight drop model is a well‐established and clinically representative model of TBI in rodents.^81^ However, this model is still relatively new in its application in the zebrafish model. Nevertheless, with some modifications toward the rodent model,^82^ this weight drop model can accurately inflict mild TBI in zebrafish.^83^ This model imitates a blow or a strike injury to the brain and gives an accurate representation of human TBI. The weight drop model in zebrafish is an efficient and cheap model that can be easily set up and conducted in any laboratory environment.

In brief, the zebrafish was fully anesthetized and was positioned with its dorsal side upward on a foam bed under the vertically erected plastic tubing. Then, the superior side of the head was properly positioned below the tube. A steel ball bearing with a mass of 0.0032 N was dropped through the plastic tube from a specified height, thereby impacting the cranium of the zebrafish at free‐fall speed and energy (Figure 4F). Finally, the fish was placed into the recovery tank. The severity of the TBI inflicted can be adjusted based on the height and weight of the ball bearing, but the consistency of these factors should be kept throughout the experiment and maybe set (marking the plastic tubing), to avoid large errors in TBI outcomes.

The selected study also summarized in Table 1, showed peak GO clusters as early as 3 days post‐injury with a peak neuro regeneration at 21 days post‐injury,^83^ which aligned with the rodent studies of this model. In addition, the MAP kinase cascade, which was activated at 3‐day post‐injury, was similarly seen in the rat weight‐drop TBI model.^84^ Similarly, behavioral assays also showed spatial memory deficits observed in other animal TBI models and human TBI patients.^85^, ^86^, ^87^, ^88^, ^89^ Taken together, the zebrafish weight drop model is an excellent, upcoming, and clinically representative model of TBI.

#### 3.3.7 Larval intracerebral hemorrhage (ICH) model

Intracerebral hemorrhage (ICH) injury accounts for about 10%–15% of strokes and about 40% of cases of disability worldwide.^90^ ICH manifests in two phases of injury; first, an influx of blood causing hematoma expansion in the brain leads to a rise in intracranial pressure surrounding the cerebral structure thereby leading to apoptosis and necrosis of neuronal cells.^91^, ^92^ The second phase includes a breakdown of blood compounds that activates the immune system and therefore, induced disruption of the blood‐brain barrier and edema development.^93^ Nevertheless, one of the mammalian studies also revealed the chronic blood‐brain barrier disruption and neuroinflammation as part of the impact after the TBI.^94^

Intracerebral hemorrhage in larval zebrafish can show spontaneous brain‐specific bleeding without any invasive techniques^95^, ^96^ and yet still able to better mimic this aspect of the human TBI condition compared to the commonly used rodent models.^97^, ^98^ In brief, atorvastatin (ATV), a known substance to cause spontaneous cerebral‐specific blood vessel rupture, was solubilized for zebrafish larvae/embryo treatment in an embryo medium‐filled petri dish (Figure 4G) and those exhibiting ICH at 24 h were separated for further analysis.^95^, ^99^, ^100^, ^101^ Some studies alternately used the "bubblehead" (bbh) mutant zebrafish line which can exhibit spontaneous ICH similar to ATV.^96^ Comparison between the two ICH protocols is yet to be discussed, but both manage to achieve similar ICH injuries in the zebrafish larval model.

The selected study showed cerebral bleeding in zebrafish larvae which led to an increase in neuronal cell death, an effect that can be similarly seen in humans^91^ but has yet to be studied in rodent models. Thus, investigating the underlying mechanism of cell death after ICH in zebrafish larvae may help uncover possible ways to inhibit the injury effect. Moreover, the study also found that motor deficiency improved at 3‐days post‐injury suggesting recovery after cell death, which may provide a good opportunity for researchers to investigate this recovery process of possible neuronal regeneration at a greater depth than the rodent model.^102^

The major advantage of utilizing this zebrafish larval ICH model is the immediate intact in vivo imaging of ICH‐induced inflammatory process upon injury induction that enables cellular interactions and signaling within the brain to be observed spontaneously post ICH, a feature not possible with mammalian models of ICH. The major disadvantage of this injury model, on the other hand, is the lack of cranium in the zebrafish larvae limits this model from developing clinically representative intracranial pressure,^92^ despite the presence of edema.^96^, ^103^ Nevertheless, this model may still be the simplest model to study ICH pathophysiology and its subsequent drug discovery.

#### 3.3.8 Larval glutamate excitotoxicity

Glutamate excitotoxicity is a secondary injury hallmark of TBI. Therefore, the larval glutamate insult injury model is a highly specific model that investigates this secondary injury pathway and may only provide therapeutic solutions targeting this pathway, as it does not cover other aspects of TBI pathology. Nevertheless, this model may provide a clear, concise, and in‐depth understanding of TBI’s glutamate excitotoxicity injury mechanism and its subsequent outcome. Besides, this injury model is a non‐invasive model since the larvae can readily absorb glutamate acid and will not be injured mechanically. The secondary injury cascade following this injury model include axonal injury, cell death and synaptic dysfunction which depends on a dose‐severity relationship.^104^

In brief, early‐stage larvae were prepared in a 96‐well plate, one larva per well^105^ (Figure 4H). On the third‐day post‐fertilization, glutamic acid was titrated in different concentrations (dose‐severity relationship), and then diluted in DMSO. Next, a confocal microscope was used to visualize the brain‐specific effects and behavioral assessment was performed via automated software that scans through the well plate.

The selected study in Table 1, showed survival curves for a dose‐dependent response to excitotoxic injury and quantifiable locomotor deficit in the injured larval, which were similarly found in TBI mammalian models of glutamate excitotoxicity.^105^ The study also suggested the model's sensitivity to detect any changes in downstream intervention therapy strategies, providing great implications for future TBI treatment studies.

#### 3.3.9 Larval stab lesion

This method induces a rapid neuroinflammatory and cellular death response similar to QA lesion but in the absence of excitotoxic agents, thus indicating this injury model to be purely mechanical. Moreover, unlike the brain mechanical lesion which requires the adult zebrafish brain to be exposed through surgical means, this larval stab lesion can be performed in the intact larvae without any incision or surgery. However, this model more closely resembles the penetrating TBI as seen in certain clinical TBI cases.

In brief, zebrafish larvae were anesthetized and placed with the abdomen facing downward onto an agarose plate. Then, by using a micromanipulator to handle the needle at a 45° angle, the needle was inserted into the hindbrain or any other intended regions, until the desired depth was reached (Figure 4I). The larvae were then released into freshwater for recovery. The injury protocol ensures the survival of the injured zebrafish larvae. Alternatively, stab lesion in the larvae can also be achieved by piercing a pin to the optic tectum of the larvae at a 20–30^o^ angle. This latter injury protocol was mainly used to investigate secondary neuronal cell death post‐injury,^106^ while the former was performed to study the brain region‐specific neuroinflammatory response after injury.^107^

The study by Gan and his colleagues (Table 1) showed that the microglial activation and expression of neuroinflammatory cytokines such as IL‐1β and IL‐6 were increased immediately following the injury, which supports previous studies on penetrating TBI in zebrafish and mice.^108^, ^109^, ^110^ Herzog's study showed that secondary cell death, caused by neuronal excitotoxicity was improved with increases in microglia phagocytosis.^106^ Both these larval stab lesion models may portray a more simple, quicker and inexpensive model to be used in understanding the diverse function of the neuroinflammatory‐cell death response, especially in terms of the role of microglial after a brain injury.^111^, ^112^, ^113^

### 3.4 Mechanism and biomarkers involved in the non‐mammalian animal model of Traumatic Brain Injury

Some regenerations signaling pathways in vivo for *C*. *elegans* on regulation of naturally occurring axon regeneration following TBI have been reported.^114^, ^115^, ^116^ Axonal regeneration is essential for the recovery process after TBI. One study previously done with APOE deficient mice/APOE mice results indicates that the Dab1‐Cdc42 pathway mediates ApoE‐induced axonal regeneration following TBI.^117^ Promoting axon regeneration is one of the therapeutic approaches following TBI. However, the CNS regeneration in mammals is still rudimentary compared to the Peripheral nervous system (PNS) and Central Nervous system (CNS) neurons in non‐mammals such as roundworm, fruit fly and zebrafish, which able to regenerate after injuries.^118^ Recently, more research on the non‐mammalian injury for regeneration models study provides tremendous opportunities to elucidate the signaling pathways that regulate naturally occurring axon regeneration.^118^

The innate immune system is highly conserved between flies and humans.^119^, ^120^ The antimicrobial peptides (AMP) gene was upregulated in the HIT model in flies, showing innate immune response pathway's activation.^39^ Toll and Imd pathways are responsible for AMP transcriptional activation.^119^ Since the flie's toll pathway is analogous with mammalian toll‐like receptor (TLR) and the immune deficiency (Imd) pathway is identical with mammalian TNF, this could help provide an opportunity to advance our understanding of this role in this fly model. Similarly, the dCHI model in flies showed increased differential expression of many AMPs, including Attacins, Cecropins and Diptericins, and Drosocin, Drosomycin, and Metchnikowin,^55^ which all regulated by the Toll, Imd, and JAK‐STAT pathways^121^ and depleted after 7 days of the injury. The short duration of genes upregulation is the same as findings in the mammalian model, where inflammatory gene spikes after TBI but dies down during subsequent days.^122^, ^123^ Autophagy pathway or intracellular clearance was also reported in the TBI fly model where previously highlighted in TBI mammalian model.^58^, ^124^, ^125^, ^126^ Atg8/MAP‐LC3 family of protein is an essential biomarker in the autophagic pathway, and there was a significant increase of Atg8a‐positive detected throughout the fly nervous system 24 h after mTBI.^10^ This study also showed increase of ubiquitinated protein and peaked at 12 h post injury^10^ which consistent with studies in controlled cortical impact mice model where a block in autophagic flux caused a build‐up of ubiquitinated proteins and p62/SQSTM 1.^58^

In the weight drop model of zebrafish, Gene Ontology (GO) discovery showed differentially expressed genes (DEG) identified in the zebrafish model after 3 and 21‐day post‐injury.^83^ Response to cAMP, which critical in neuronal survival, is one of few significant GO clusters found. Also seen is the presence of the mitogen‐activated protein (MAP) signaling pathway where Jun N‐terminal kinases (JNKs) within the MAP kinase family helps in JunB protein phosphorylation which is important in tissue regeneration of zebrafish.^127^ This event is the same as found in the mild fluid percussion TBI model in rats, the increase of Junb expression, ipsilateral to the injury site was previously reported.^128^, ^129^ Next, Notch 1b which required for neurogenesis also seen upregulated after injury.^83^ Notch 1 signaling has shown to promote production of Neural progenitor cells (NPC) that migrated toward the damage site.^130^, ^131^

Essential mature neuronal biomarkers such as MAP2a+b, parvalbumin, SV2, and metabotropic glutamate receptors 2 (mGLU2) were also identified in the zebrafish TBI model,^74^ and these suggested that newly generated neurons differ from the mature neurons. On the other hand, in mammals, radial glial cells transform into multipolar astrocytes, which is no longer exist in the radial oriented cell.^132^ In mice, activated astrocytes become hypertrophic within few days, as it will upregulate to intermediate filament Glial fibrillary acidic protein GFAP, Nestin, and Vimentin as well as chondroitin sulfate proteoglycans, Tenascin C and other extracellular matrix (ECM) components.^133^, ^134^, ^135^ One of the TBI models conducted in zebrafish proposed the importance of the specific glial environment for long‐term neuronal survival.^77^ Besides that, small injury showed different when there is less, or almost no radial glial like cell surround the injury, which in contrast to strong agliosis which assemble around the stab wound injury in rodent telencephalon.^77^

The radial glial cell is known to have a strong ability to generate neurons.^136^, ^137^, ^138^, ^139^ Increase of radial glial cells expression on GFAP‐GFP after 3 days of injuries in zebrafish was seen. This event also similarly found in adult neurons of the mouse brain after stab lesion^133^ which suggested the conserved response on the GFAP expression to stab injury in mammalian and teleost. In injured zebrafish, the lesioned hemisphere showed olig2: EGFP positive cells at the lesion site. Transcription factor of Olig2 is expressed in mature oligodendrocyte and Oligodendrocytes and their progenitor cells (OPCs) in zebrafish and mouse.^140^, ^141^, ^142^, ^143^ Olig 2‐EGFP transgenic line acts as a reporter of Olig2 expression showed only a temporary effect in zebrafish. Indeed, the proliferation of Olig 2 expressing population of mature oligodendrocyte and OPC is relatively moderate after injury in zebrafish compared to in mammals, where OPC is highly reactive in brain injury and promotes brain glial scar.^144^, ^145^, ^146^, ^147^ Zebrafish do not exhibit continuous inflammation and do not have any astroglial scarring compared to in mammal CNS.^77^ This glial scarring was said to hinder the repair of brain cells in mammalian model as reviewed previously.^133^, ^134^, ^148^, ^149^ Hence, the lack of later stage of inflammatory response in zebrafish leads to the successful neuronal repair of these animal model. However, the damping inflammation process in zebrafish also remains unknown.

In mammals, brain injury stimulates cell proliferation and neurogenesis in the Subventricular zone (SVZ).^150^, ^151^, ^152^, ^153^ While in zebrafish, Notch 1 plays a vital role in the proliferation and neurogenesis of zebrafish identified in the Ventricular zone of the zebrafish's injured hemisphere.^130^ The study identifies the subpopulation of her4.1 expressing Radial glial progenitor cell as the main neurogenic population reacts to the lesion and as the primary source of newly generated neurons.^74^ In mice, Angiopoietin (Ang1) and Stromal‐derived factor 2 (Sdf1) are responsible for regulating SVZ Neural progenitor cell migration in mice after stroke^154^ but not reported in adult zebrafish. Prokineticin 2 (PROK2), a chemokine that guides the migration of SVZ‐Derived NPC toward olfactory bulb in mammals^155^, ^156^ is also found in zebrafish.^157^ PROK2 may become an essential biomarker for both constitutive and injury‐induced adult neurogenesis in the zebrafish brain. Gata 3 expressed after the zebrafish brain injury and involved in the early role in zebrafish brain regeneration^158^ and Fibroblast Growth Pathway (FGF) signaling pathway may directly affect the expression of Gata 3 after injury in zebrafish tissues.

Quinolinic Acid (QA) injury in zebrafish^72^ showed an increase in microglial reaction, NPC proliferation and damage repair. NPC radial glial cells reported generating beneficial neuronal subtypes such as neurons that give rise to the long‐distance projection that bridges the synaptic connection with the contralesional hemisphere. QA direct action on glutamate receptors, also involved in mammals’ neurogenesis.^159^ Besides that, a study on transgenic fish in discovered the new role of the sigma‐1 receptor in modulating microglia responses to brain injury and propose new further investigation on this receptor to prevent further chronic neuroinflammation.^160^

### 3.5 Future direction of non‐mammalian animal model in Traumatic Brain Injury research

Roundworm, fruit fly, and zebrafish been utilized as an animal model of traumatic brain injury are still new and not much research till recently. Most of the reported studies showed several biomarkers, and underlying mechanisms for the establishment of a disease model. As a TBI animal model, *Caenorhabditis elegans* showed a reduction in mobility, paralysis, and morphological changes,^27^, ^34^ which can help in TBI's future research, especially in the early phase a study. Those researches could benefit from these animal models where the disease study can be done quickly. Indeed, a high amount of results can be produced within a specified period.

On the other hand, *Drosophilia melanogaster* can be the best candidate for the first screening test to test the important pathways that modulate recovery. Drosophila model showed a strong upregulated immune response shortly after TBI. Immune and stress response make up 157 out of 512 different glial genes.^55^ Simultaneously, genes for proteolysis and protein folding are the major portion (85/512) of these differentially expressed genes.^55^ Besides, sleep cycle impairment after TBI has also been studied by using this closed‐head TBI model where Nuclear Factor kappa B (NF‐κB), Dopamine transporter (DAT), and Pale (ple) played a critical role in affecting this sleep cycle.

*Danio rerio* was reported to offer a wide range of opportunities to study inflammatory response and successful regeneration programs in the Central Nervous System.^161^ Thus, help in the search for potential therapeutic applications for TBI and neurodegenerative disorders. Nevertheless, current technology developed a highly sophisticated imaging system used in zebrafish TBI studies.^162^ This helps to provide a detailed understanding of the brain after the injury and can be further characterized.

Apart from that, the wound healing related pathway process reported with the interaction between inflammation, neurogenesis, and angiogenesis has been characterized in this study.^75^ This system can provide a basis for analyzing high‐throughput data, a promising way to discover TBI biomarkers. In addition to that, the non‐mammalian model can provide an important biological basis for regeneration studies and can be used to test potential therapies that might enhance the regeneration process in the mammalian model.

Not to forget, the unique Danio rerio larvae, with undeveloped blood‐brain barrier^105^, ^107^ it can absorb neuroprotective drug directly and allow greater bioavailability in determining the drug effectiveness. This model describes the secretion of the pro‐inflammatory cytokine IL‐1B, IL‐6 by M1 type microglia, and anti‐inflammatory factors include IL‐4 and IL‐10 by M2 type microglia.^107^ Hence, this model can use as an in vivo system tool to study the different functions of activated microglia and for screening chemicals for CNS disorder‐related diseases. Morphological changes such as axonal blebbing and fragmentation of degenerating axons can be seen in this animal model following the brain injury, which has been previously described.^105^

All the non‐mammalian model has a great potential to expedite the stream of drug discovery for the TBI treatment. Ongoing research of TBI with a non‐mammalian model is often compared with the current established mammalian model. Both models are essential in TBI research, where non‐mammalian helps in screening for the important gene expression involved, biomarkers, and mechanisms for TBI research due to its simple nervous system and highly reproducible. On the other hand, the mammalian TBI model is more advanced in its robust research background focusing on the mechanism and important biomarkers.^117^, ^163^, ^164^, ^165^, ^166^, ^167^, ^168^ In addition to that, non‐mammalian research has not conducted any research on inducing stem cell for brain injury as been conducted previously in this study with mammalian model.^169^, ^170^ Indeed, one of the study showed successful xenotransplantation of human iPSC‐derived NSCs and isogenic neural cell progenies in a mouse model.^171^ Several of the limitations on mammalian model such as the sample size being often small and bear a high cost, often hindered the ongoing research. Hence, the non‐mammalian model could be utilized initially on the early phase of study. Genetic approaches are not the same for both mammalian and non‐mammalian. So, it is not practical to perform large‐scale screening on rodents only to identify the important signaling mammalian pathways. Hence, to demonstrate the pathways in non‐mammalian models which also has the functions in non‐mammalian model could be a great focus in the future studies. Nevertheless, both models contribute to a beneficial future of TBI research.

## 4 CONCLUSION

In summary, we believe this is the first review that summarizes and evaluates non‐mammalian models of TBI while critically comparing them with mammalian models of TBI, in hopes to elucidate a better, cheaper, faster, and more efficient alternative TBI model that may further advance current TBI research. Among the three species of non‐mammalian TBI model retrieved in this review, comprised of zebrafish, roundworm, and fruit fly, zebrafish is the one most frequent model used in showcasing a variety of injury conditions to mimic human TBI. Moreover, since both the larvae and adult zebrafish can be effectively sampled to inflict clinically representative TBI, whether penetrating or closed head injury, an endless possibility of TBI investigations such as aging effects, intervention studies, and time progression outcomes, can be performed with hassle‐free. On the other hand, the roundworm and fruit fly are mostly ideal for high‐throughput TBI treatment screening studies. They may be limited in terms of pathophysiological resemblance to human TBI, despite the similarities in some of the functional outcomes displayed post‐injury when compared to mammalian TBI models and human TBI. Taken together, the non‐mammalian TBI models provide a more simplistic approach to bridge the knowledge gaps within TBI research and may shorten the road toward TBI outcome prevention and cure, by surpassing the limitations of mammalian models of TBI. Apart from that, these animal models could be used together with the mammalian model and help on the trial basis of research before moving to an in‐depth investigation to find the novel mechanism and therapeutic approaches against TBI.

## CONFLICT OF INTEREST

All authors declare that they have no conflict of interest.

## AUTHOR CONTRIBUTIONS

NAZ performed literature search, analyzed, and wrote the manuscript. AA contributed to quality analysis, writing, and editing. MFS conceptualized the idea, designed, and supervised the work. AA, IA, SAZA, IO and MFS provided their critical comments and were involved in editing and proofreading. All authors gave their final approval prior to the submission of this manuscript.

## Supporting information

Table S1

## Data Availability

Data sharing is not applicable to this article as no new data were created or analyzed in this study.
