# Supplementary material for: The utilization of small non‐mammals in traumatic brain injury research: A systematic review
Source: CNS Neurosci Ther. 2021 Feb 4;27(4):381–402. doi: 10.1111/cns.13590 (PMC7941175; doi:10.1111/cns.13590)
Supplement: Supplementary file 1 — Table S1 [file CNS-27-381-s001.docx]

Table S1: SYstematic Review Centre for Laboratory animal Experimentation Risk of Bias (SYRCLE RoB tool)

| No | Studies | Was the allocation sequence adequately generated and applied? | Were the groups similar at baseline or were they adjusted for confounders in the analysis? | Was the allocation adequately concealed? | Were the animals randomly housed during the experiment? | Were the caregivers and/or investigators blinded from knowledge which intervention each animal received during the experiment? | Were animals selected at random for outcome assessment? | Was the outcome assessor-blinded? | Were incomplete outcome data adequately addressed? | Are reports of the study free of selective outcome reporting? | Was the study free of other problems that could result in a high risk of bias? |
| --- | --- | --- | --- | --- | --- | --- | --- | --- | --- | --- | --- |
|  |  | **1** | **2** | **3** | **4** | **5** | **6** | **7** | **8** | **9** | **10** |
| 1 | (Angstman et al., 2018) | U | Y | U | Y | N | N | N | Y | Y | Y |
| 2 | (Angstman et al., 2015) | U | Y | U | Y | N | N | N | U | Y | Y |
| 3 | (Miansari et al., 2019) | U | Y | U | Y | N | N | N | U | Y | Y |
| 4 | (Hockey et al., 2013) | U | Y | U | Y | N | N | N | U | Y | Y |
| 5 | (Sun & Chen, 2017) | U | Y | U | Y | N | N | N | U | Y | Y |
| 6 | (van Alphen et al., 2018) | Y | Y | U | Y | N | N | N | U | Y | Y |
| 7 | (Katzenberger et al., 2013) | U | U | U | Y | N | Y | N | U | Y | Y |
| 8 | (Putnam et al., 2019) | U | Y | U | Y | N | Y | N | U | Y | Y |
| 9 | (Katzenberger, Loewen, et al., 2015) | U | Y | U | Y | U | Y | U | U | Y | Y |
| 10 | (Katzenberger et al., 2016) | U | Y | U | Y | U | Y | U | U | Y | Y |
| 11 | (Barekat et al., 2016) | Y | Y | N | Y | N | N | N | U | Y | Y |
| 12 | (Ferrier et al., 2017) | U | U | U | Y | U | Y | U | Y | Y | Y |
| 13 | (Lim et al., 2016) | Y | Y | U | Y | U | Y | U | Y | Y | Y |
| 14 | (McCutcheon et al., 2016) | U | Y | U | Y | U | U | U | Y | Y | Y |
| 15 | (Crilly et al., 2018) | Y | Y | U | Y | Y | Y | Y | Y | Y | Y |
| 16 | (McCutcheon et al., 2017) | U | Y | U | Y | Y | Y | Y | Y | Y | Y |
| 17 | (Skaggs et al., 2014) | U | Y | U | Y | U | Y | U | Y | Y | Y |
| 18 | (Gan et al., 2019) | U | Y | U | Y | U | Y | U | Y | Y | Y |
| 19 | (Herzog et al., 2019) | Y | Y | Y | Y | Y | Y | Y | Y | Y | Y |
| 20 | (Diotel et al., 2013) | U | Y | U | Y | U | U | N | Y | Y | Y |
| 21 | (Zhang et al., 2015) | U | U | U | Y | U | U | U | Y | Y | Y |
| 22 | (Kishimoto et al., 2012) | Y | Y | U | Y | U | U | U | Y | Y | Y |
| 23 | (Wu et al., 2014) | Y | Y | Y | Y | Y | Y | Y | Y | Y | Y |
| 24 | (Ayari et al., 2010) | U | Y | U | Y | U | Y | U | Y | Y | Y |
| 25 | (Kroehne et al., 2011) | U | Y | U | Y | U | Y | U | Y | Y | Y |
| 26 | (März et al., 2011) | U | Y | U | Y | U | U | U | Y | Y | Y |
| 27 | (Schmidt et al., 2014) | U | Y | U | Y | U | U | U | Y | Y | Y |
| 28 | (Baumgart et al., 2012) | U | Y | U | Y | U | Y | U | Y | Y | Y |
| 29 | (Maheras et al., 2018) | U | Y | U | Y | Y | Y | Y | Y | Y | Y |

Abbreviations: N, No; Y, Yes; U, unclear
